# Postoperative Acute Pancreatitis After Pancreatic Resections—A Narrative Review and a Diagnostic Algorithm

**DOI:** 10.3390/cancers17172773

**Published:** 2025-08-26

**Authors:** Ewa Grudzińska, Magdalena Gajda

**Affiliations:** Department of Gastrointestinal Surgery, Medical University of Silesia, 40-752 Katowice, Poland; kchpp@sum.edu.pl

**Keywords:** pancreatic cancer, pancreatitis, pancreatic fistula, pancreatoduodenectomy

## Abstract

Pancreatic cancer is one of the deadliest tumors, and surgery is, for now, the only potentially curative treatment. However, pancreatic surgery is burdened with severe complications, of which postoperative pancreatic fistula (POPF) is one of the most dangerous. Recent publications show that there is a strong connection between POPF and postoperative acute pancreatitis (POAP). In this review, we present the available literature on PPAP, summarizing the available diagnostic, prophylactic, and treatment tools. We identify POAP as a specific postoperative complication where a lack of unified definitions and treatment guidelines makes both research and clinical decisions more difficult. Based on the available studies, we also propose an algorithm for early POAP detection.

## Simple Summary

One of the most severe complications after pancreatic surgery is postoperative pancreatic fistula (POPF), directly associated with postoperative acute pancreatitis (POAP). Currently, there is no international consensus on POAP classification, diagnostics, treatment, and prophylaxis. In this review, we present the available literature on the topic, and for clinical purposes, we propose a diagnostic algorithm for POAP detection.

## 1. Introduction

Pancreatic cancer is one of the leading causes of cancer-related death. According to epidemiological data, most patients with pancreatic cancer have distant metastases already in the asymptomatic phase of their illness. Five-year survival is estimated at only 11%, and morbidity almost equals mortality. Surgery is the only potentially curative possibility in pancreatic adenocarcinoma; however, it has a high risk of complications [1].

One of the most severe complications is postoperative pancreatic fistula (POPF), which is found in 10–15% of patients after pancreatoduodenectomy (PD) and in 10–30% of patients after distal pancreatectomy [2]. It harms overall survival; it can cause postponement of adjuvant therapy, prolongation of hospitalization, and increase treatment costs [3,4,5]. Mortality due to POPF can reach 5.4%. Recent publications suggest that postoperative acute pancreatitis (POAP) can be essential in the development of POPF [6,7]. The postpancreatectomy acute pancreatitis has been recently distinguished from other cases of postoperative pancreatitis as PPAP [8]. In the following manuscript, we mostly use the term “POAP”, as we did not want to alter the terminology used by the cited authors.

In this review, we aim to summarize the available studies on the POAP diagnostic criteria and classifications, and based on these, create a simple diagnostic algorithm useful in a clinical setting that may enable early diagnosis of POAP. We also wish to identify areas where further research and formulation of clinical guidelines are needed to improve pancreatic surgery outcomes.

We wish to emphasize that this is a narrative review; therefore, the PRISMA methodology was not applied, which may have caused a selection bias. However, we did our best to perform an extensive literature search using PubMed and Google Scholar (with keywords like “POAP”, “PPAP”, “postoperative acute pancreatitis”, “pancreatoduodenectomy complications”, “acute pancreatitis treatment”, “postoperative pancreatitis treatment”, etc.) and include the most relevant publications.

## 2. POAP Pathophysiology

**Figure 1:**
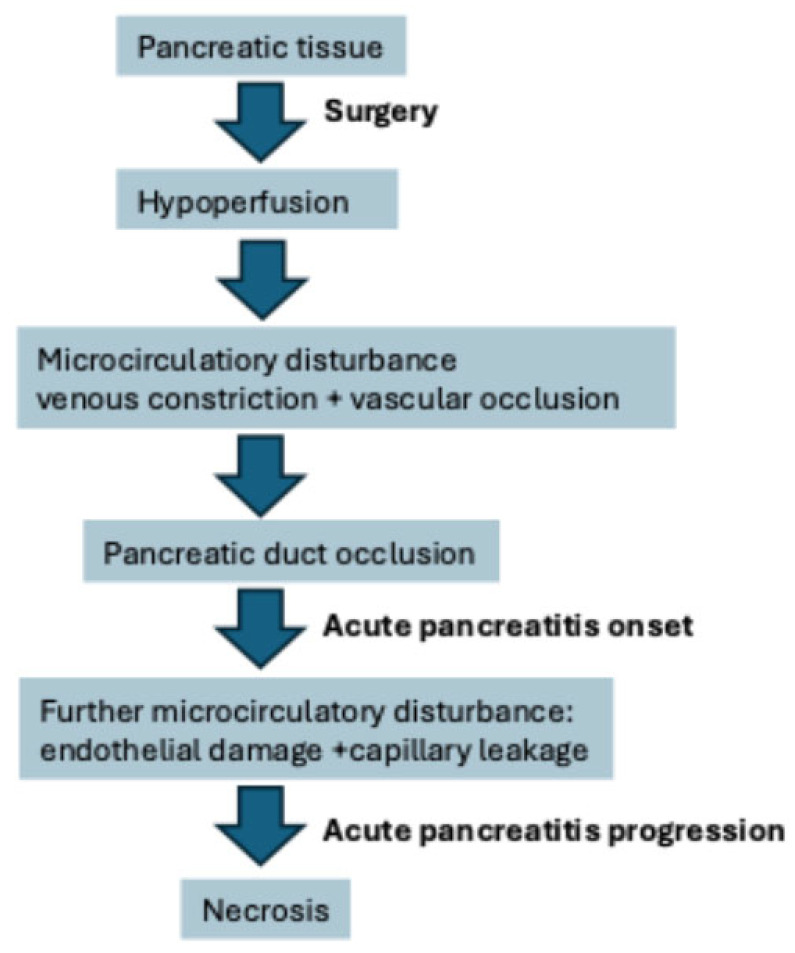
POAP pathophysiology [7,10].

POAP most often occurs after pancreatic surgery (1.9–50%) with up to 80% mortality [6], but it can also develop occasionally after gastric or esophageal surgery due to unintended injury of the pancreas [5,9]. In 2016, Connor suggested that POAP causes a large portion of POPF and that POAP is a new specific complication of pancreatic surgery [7]. According to this author, the main reason for POAP is pancreatic ischemia (Figure 1). In a healthy organism, the whole gland is perfused equally, with blood flow strictly bound to pancreatic excretion. Hormonal (somatostatin, secretin, cholecystokinin) and neuronal autoregulation allow for sufficient pancreatic perfusion if the blood flow is at least 40 mL/min/100 g of pancreatic tissue [10]. In mild pancreatitis, slight hyperemia of the organ is found with elevated oxygen pressure in the tissue and elevated hemoglobin oxygen saturation. However, as the disease progresses, lower perfusion occurs, leading to pancreatic ischemia and promoting inflammation. This, in turn, causes tachycardia, a decrease in blood pressure, a lower cardiac index, and a decrease in central venous blood pressure. This central instability is exacerbated by portal stasis, further obstructing pancreatic perfusion and causing blood stasis in capillary vessels.

Even when aggressive fluid resuscitation is administered to ameliorate the cardiac output, the pancreatic perfusion remains insufficient because of changes in microcirculation, causing the pancreatic perfusion to decrease and be disproportionate to the decrease in cardiac output. It has been shown that severe acute pancreatitis is associated with decreased tissue perfusion and lower blood oxygen pressure in the pancreas [11,12]. Further studies suggest that the blood flow in severe pancreatitis is insufficient to remove enzyme degradation products, which may lead to the aggravation of the illness [11,13,14].

Microvascular changes play a great role in acute pancreatitis etiology [15]. As early as 6 min from the onset of acute pancreatitis, the pancreatic blood flow, red blood cell density, and velocity decrease, becoming even more evident 8 h from the beginning of the illness. The number of perfused capillaries also significantly decreases [14,16]. As was shown in animal models, the progressive exclusion of the capillaries starts within the first 30 min of pancreatitis onset and causes their almost complete destruction in 3 h. These changes are present where the most severe illness is found, while the less affected regions of the pancreas present dilated vessels and hyperemia [17]. The total blood flow in the pancreas does not correlate with the regional perfusion, as the fenestrated pancreatic capillaries contribute to blood stasis. The pancreatic anastomosis dehiscence following POAP may result from pancreatic stump ischemia and necrosis [18]. If POAP does not subside, it may also lead to intra-abdominal abscesses, hemorrhage, infection, and even death [18].

In the POAP pathophysiology, the role of systemic inflammatory response must also be mentioned. In the course of pancreatitis, the increase in intracellular calcium levels and oxidative stress initiates an inflammatory cascade. The local inflammation often progresses to a systemic inflammatory response (SIRS), with abundant release of proinflammatory cytokines and other mediators (tumor necrosis factor, thromboxane A2, platelet-activating factor, endothelin, interleukin-1 and -6). The intestinal barrier failure and the leakage of toxic factors (endotoxin, phospholipase A2) amplify the inflammatory response. A premature zymogen activation in the pancreas induces “self-digestion” of the surrounding tissue, and trypsin is released to the bloodstream, stimulating macrophages and increasing cytokine release. Furthermore, in the course of severe acute pancreatitis, acute lung injury may occur, leading to respiratory failure. Consequently, in POAP, multiorgan failure is not rare, and the patients often require multidisciplinary intensive care [19,20].

## 3. Risk Factors for POAP

In a study by Ikenaga, risk factors of POAP differed between PD and distal pancreatectomy (DP). In patients after PD, the risk of POAP correlated with longer operative time (>295 min), soft pancreatic tissue, and main pancreatic duct (MPD) < 3 mm. For DP, the risk factors included ASA ≥ 2 (American Society of Anesthesiologists physical status classification) and pancreatic thickness ≥ 9.5 mm [21]. In a recent study, Ji et al. assessed preoperative inflammatory markers in PD patients and their association with postoperative complications. They found that high preoperative interleukin-6 levels are associated with a higher risk of POAP [22].

A study by Kühlbrey [23] also showed a difference between the surgery type groups and in patients after DP, unlike in the PD group, serum amylase activity did not correlate significantly with POPF formation.

Chen [24] examined patients after PD. 770 (52.56%) had POAP (defined by Connor’s criteria). In a multifactorial analysis, female sex, normal preoperative bilirubin level, robotic surgery, non-dilated MPD, and a diagnosis other than adenocarcinoma (e.g., cystic or neuroendocrine tumors) were independent predictive factors for POAP. MPD without dilatation had a sensitivity of 72.16% and specificity of 71.84% in POAP prediction. Of 277 patients with clinically relevant POPF (CR-POPF), in 221 (79.78%), POAP was also found. The author observed that robotic PD was an independent risk factor for POAP and a predictive factor for CR-POPF. The CR-POPF rate was similar between open and robotic PD (19.54% vs. 17.27%), but POAP was more prevalent in robotic surgery (66.42% vs. 47.15%).

In multifactorial analysis by Andrianello [25], pancreatic tissue sparing DP, pancreatic thickness, and neuroendocrine tumors were independent risk factors of POAP. On the contrary, neoadjuvant therapy, age over 65, and dilated MPD were independently connected to lower POAP risk.

These data show that risk factors for POAP differ depending on the type of surgery. Probably, the closure of the pancreatic stump after DP is less dependent on the healing processes and more on the mechanical stability. On the contrary, POPF formation after PD seems more complex and dependent on the local inflammation status rather than the mechanical effect of the sutures. Of the modifiable factors, the reduction in the operative time and neoadjuvant therapy administration may be considered to reduce the POAP rate. In Table 1, we present a summary of the POAP risk factors.

## 4. POAP Diagnostics

### 4.1. Diagnostic Criteria and POAP Classification

Acute pancreatitis is diagnosed based on clinical symptoms, physical examination, and laboratory results.

Two of these three criteria must be fulfilled according to the Atlanta classification:

(1) Acute epigastric pain, often radiating to the back;
(2) Lipase or amylase levels in serum elevated more than 3 times above the normal limit;
(3) Imaging tests typical for acute pancreatitis (computed tomography—CT, magnetic resonance imaging—MRI, or abdominal ultrasound).

For a long time, POAP had the same diagnostic criteria. It is agreed that serum activity of the pancreatic enzymes is a proper criterion of POAP. Epigastric pain, however, is commonly seen in patients after pancreatic surgery, which in clinical practice reduces its usefulness as a POAP symptom. The imaging results are doubtlessly helpful in postoperative diagnostics, but very tricky to interpret. PPAP usually shows irregular fatty tissue enhancement and inflammatory changes in the postoperative site, around the pancreatic stump. Such images may be confused with postoperative changes, especially in milder cases of POAP [26]. Abdominal CT is, therefore, a useful diagnostic tool, but requires much experience, especially in early POAP stages.

Because the above classification did not seem entirely appropriate for POAP, some authors used Connor’s classification, which includes serum markers, urine trypsinogen-2 (u-TRP), drain amylase activity, and clinical condition of the patient (i.e., abdominal pain, distention, impaired bowel function, fever, DGE) [7,18]. The classification includes a clinically relevant POAP (CR-POAP) when C-reactive protein (CRP) is elevated above 180 mg/L on the second postoperative day (POD) (Table 2). The serum and urine biochemical POAP markers are present already on the first POD. In an observational study of 61 patients after pancreatic resection, the presence of POAP on the first postoperative day, as defined by these markers, was also a strong predictor of POPF (OR 17.81, 95% CI 2.17–145.9) [27].

A strength of Connor’s classification is its arrangement of POAP grades based on the ISGPS POPF grades, ensuring data clarity and a direct point of reference. Furthermore, it precisely defines the postoperative days during which individual laboratory parameters should be measured. However, this classification does not address the condition of the pancreatic parenchyma or the presence of histological changes such as necrosis, which may indicate a weakness of this system. Also, it includes the drain amylase activity as one of the criteria; however, amylase presence in drains is rather studied as a sign of POPF and not necessarily POAP.

Another classification of POAP was proposed by Globke [28], which added postoperative acute necrotizing pancreatitis (POANP) as an additional class. The biochemical criteria are the same as in the Atlanta classification. The author analyzed two groups of patients after pancreatoduodenectomy (PD) followed by total pancreatectomy. Necrotic POAP (POANP) was found in histological examination in 33 (41%) of 79 patients. 46 (58%) of patients had POAP without histological evidence of necrosis in the removed pancreatic stump. The CRP serum concentrations on POD2 and on the day of reoperation were significantly higher in the POANP group. Also, APACHE II (Acute Physiology and Chronic Health Evaluation II) and SOFA (The Sequential Organ Failure Assessment) grades assessing the severity of general clinical condition were higher in POANP patients after the first surgery. POANP patients had longer intensive care and hospital stay. Also, Clavien-Dindo grade ≥ 3 (severe complications) and 30-day reoperation rate were significantly more prevalent in the POANP group. The Globke classification differentiates POAP types based on the presence of necrosis in the pancreatic parenchyma, while its biochemical criteria are based on the Atlanta classification. This appears to be insufficient, as the Atlanta classification refers only to elevated pancreatic enzyme activity levels without specifying a specific time for their measurement. Clinical use of this system would require refinement of these data.

The PPAP was introduced as the main terminology for postpancreatectomy pancreatitis in 2022. Realizing the lack of a universally accepted definition, the International Study Group on Pancreatic Surgery (ISGPS) published its definition and grading [8], differentiating it from the POAP, which may occur after other types of surgery. By this definition, the disease onset should occur during the first three postoperative days, and three factors are required for the diagnosis: 1—hyperamylasemia for at least 48 h postoperatively, 2—clinically relevant features, and 3—radiologic features of PPAP. The grading is analogous to that of the POPF, with grade A—only biochemical changes, B—mild/moderate complications, and C—severe complications. Other than in Connor’s classification, the authors did not include drain amylase, considering elevated drain amylase a sign of POPF rather than PPAP. This seems justified, as the presence of pancreatic enzymes in the abdominal fluid postoperatively most probably indicates anastomotic dehiscence, which may be caused by PPAP, but may also occur independently. Also, uTRP-2 was omitted, which is probably due to its low availability in the clinical setting.

### 4.2. Serum Amylase, Lipase, and CRP

Multiple processes in pancreatic cells are reflected in biochemical changes in the blood. Rudis [6] examined 160 patients after PD due to pancreatic adenocarcinoma. In 7 patients, POPF caused death, and in 4 of these patients, POAP was found on autopsy. The authors compared these four patients’ laboratory results with 10 patients who had POPF grade C without POAP and 12 patients without any complications. In the group with POAP coexisting with POPF, serum amylase was significantly higher on POD 1 and 3 than in the other two groups. The authors also observed that a significant increase in amylase and CRP in serum is a marker of POAP development.

Many authors mention CRP as the most reliable parameter reflecting the development and course of POAP [6,28,29]. In the study by Globke [28] mentioned above, patients who after PD underwent reoperation with total pancreatectomy due to acute necrotic pancreatitis of the pancreatic stump had a much higher CRP median serum concentration on POD 2 after the PD (190 (130–241) vs. 130 (88–164)) and on the reoperation day (226 (186–260) vs. 139 (107–185)) than the patients without necrotic pancreatitis. Birgin [30] examined 190 patients after PD. In 100 (53%), POAP was diagnosed with the use of Connor’s criteria. In 35 of these patients, a clinically relevant POPF was found (22 grade B and 13 grade C). Elevated serum CRP on POD 2 and elevated serum lipase on POD 1 were connected to the onset of clinically relevant POAP. Lipase concentration of 924 U/L on POD 1 (sensitivity 63%, specificity 78%, positive predictive value (PPV) 54%, negative predictive value (NPV) 78%; *p* = 0.004) was identified as a useful threshold value to stratify the patients with a developing clinically relevant POAP (AUC 0.680 [0.568–0.793]). The serum CRP of 150 mg/L on POD 2 (sensitivity 71%, specificity 52%, PPV 45%, NPV 77%; *p* = 0.034) (AUC 0.629 [0.512–0.746]) was a good predictor of the onset of the complication’s clinical relevance in a group of patients with POAP.

Partelli [31] created a modification of Connor’s definition, based on CRP level. According to Connor, clinically relevant POAP (CR-POAP) is defined as serum amylase above normal (>100 U/L) on POD 0–1 and CRP above 180 mg/L on POD2. The author proposed a modified definition of clinically relevant POAP (m-CR-POAP) as a 3-fold elevation of serum amylase in POD1 with CRP > 180 mg/L on POD2. He analyzed 610 patients after PD and found that 150 (25%) had a clinically relevant POAP according to the former definition, and 72 (12%) had m-CR-POAP. Among patients with CR-POAP, in 74 (49%), CR-POPF was diagnosed. Serum amylase of 109 U/L on POD1 was identified as an optimal value for CR-POPF prediction, with 74% sensitivity and 71% specificity. Generally, the highest serum amylase activity is found in all patients on POD 1 after PD. In this study, in 360 patients (59%), serum amylase did not exceed 100 U/L, 142 (23%) had amylase > 100 U/L but ≤300 U/L, and the remaining 108 patients (18%) had serum amylase > 300 U/L.

Ikenaga [21] also analyzed different serum amylase activities on POD1 in the diagnosis of POAP and CR-POPF. CR-POAP was diagnosed when CRP was above 180 mg/L on POD3, which is a modification of Connor’s CR-POAP definition (where CRP ≥ 180 mg/L on POD2 was included).

Kühlbrey [23] performed a large study of 739 patients after PD or DP. They were divided into groups according to their amylase levels on POD1:

- 0–12 U/L—low;
- 13–53 U/L—normal;
- 54–158 U/L—elevated but not POAP;
- above 159 U/L—POAP (as in Atlanta definition, amylase concentration at least three times normal).

A total of 471 patients had elevated amylase. Also, 256 patients (35%) on POD1 had POAP, and 39% of these patients developed a POPF grade B or C. CR-POPF occurred in 21% of patients with elevated amylase but less than three times normal and in 9% of patients with normal amylase. No patient in the low amylase group developed POPF. The authors noted that the value of postoperative serum amylase in POPF prediction does not improve but decreases with time. This suggests that the leak in the anastomosis occurs promptly after surgery, and the amylase level may return to normal despite fistula formation.

Andrianello [25] also tried to define the predictive factors for POAP. In his study, POAP was found in 67.9% of patients (*n* = 250) and CR-POAP in 45.1% (*n* = 166). Patients with POAP had a statistically higher drain amylase on POD1 and higher CRP on POD2 and 3.

The latest research suggests that isolated amylase increase (postoperative hyperamylasemia—POH) does not necessarily indicate POAP development. It was postulated by Loos [32], who showed that POH is frequent after PD. He analyzed 1235 patients after PD. Hyperamylasemia was defined as serum amylase activity higher than normal, but also three times normal. Radiological criteria were added to the definition of POAP to differentiate it from hyperamylasemia. POAP was found in 14.7% of the cohort of 1235 patients (95% CI, 13.4–16.1%) and 29.2% (95% CI, 27.5–31.0%) of the patients with isolated hyperamylasemia on POD1. Hyperamylasemia (amylase > 159 U/L (>three times normal serum amylase activity) on POD1 and CRP > 135 mg/L on POD2 had the best effectiveness and was most practical for POAP diagnosis in the study.

Other inflammatory markers of POAP are also investigated. Aronen et al. recently found that plasma soluble urokinase plasminogen activator receptor level on POD 1 is significantly lower in patients with POPF and POAP; however, this marker is not generally available for testing [33].

### 4.3. Urine Trypsinogen-2

Trypsinogen-2 is one of two trypsinogen isoenzymes. In healthy people, trypsinogen-1 has lower kidney reabsorption than trypsinogen-2, so it is found in higher concentrations in urine. In patients suffering from acute pancreatitis, a selective increase in trypsinogen-2 is observed in serum and urine, making this isoenzyme a useful marker of the disease. U-TRP-2 has high specificity and sensitivity in acute pancreatitis diagnosis, regardless of the etiology. U-TRP-2 > 50 μg/L on POD1–2 is one of the POAP diagnostic criteria in Connor’s definition [7]. A strip immunochromatography test was invented to quickly assess u-TRP [34]. Räty applied a u-TRP strip test in POAP diagnosis [35] and examined 55 patients after pancreatic resections, performing the strip test on POD 1–7. It was positive in all 13 patients with POAP, and in twelve, it was positive already on POD1. Its sensitivity, specificity, PPV, and NPV were 100, 92, 81, and 100%, respectively. Patients who had POPF had positive strip tests more often than patients without POPF.

### 4.4. Drain Fluid Amylase

Elevated amylase and/or lipase in the abdominal drain fluid may not only be a POPF but also a POAP predictor. A threefold increase above normal serum level in drain amylase is included in Connor’s POAP definition. The available publications presented below are on POPF; we have not found any studies on drain amylase concentrations in POAP.

De Reuver [36] and Nahm [37] showed that high amylase activity in abdominal fluid obtained intraoperatively from the peripancreatic region (called intraoperative amylase concentration, IOAC) is highly predictive for POPF development after PD and DP.

The area under the receiver operating characteristic (AUROC) for IOAC as a POPF predictor was 0.93 (95% CI 0.87–0.99) for PD and 0.92 (95% CI 0.81–0.99) in DP. Molinari [38] established that drain amylase ≥ 5000 IU/L is the threshold value for POPF diagnosis, and Facy [39] found that drain lipase of 1000 IU/L had 93% sensitivity and 77% specificity in CR-POPF diagnosis. In a recent study by Chang et al., in a group of 6087 patients, the threshold drain amylase activity on POD1 after PD was set at 720 IU/L for POPF diagnosis [40].

Kühlbrey [23] in an analysis of 739 consecutive patients after pancreatic resections stated that elevated drain amylase on POD1 was present in patients developing pancreatic fistulas. Jin [41] examined the predictive value of gastric/pancreatic amylase ratio (GPAR) on POD 3 in 61 PD patients. AUROC was 0.955 (95% CI 0.87–1; *p* < 0.001) with 90.5% sensitivity, 100% specificity, PPV 100%, and NPV 95.1%. Testing GPAR on POD3 allowed for correct POPF prediction in 19 of 21 patients who developed POPF. High GPAR values and cloudy fluid in the postoperative drains were shown to be POPF risk factors.

Partelli [42] analyzed 463 patients after PD. Postoperative morbidity and mortality were 58% and 4%, respectively. In 64 patients (14%), CR-POPF was diagnosed. Multifactorial analysis identified male sex (OR 2.29 95% CI: 1.12–4.70, *p* = 0.023), drain amylase on POD 1 > 500 U/L (OR 21.72, 95% CI: 7.41–63.67; *p* < 0.0001), CRP on POD2 > 150 mg/L (OR 3.480, 95% CI: 1.21–9.99, *p* = 0.021), and CRP on POD 3 > 185 mg/L (OR 6.738, 95% CI: 1.91–23.78; *p* = 0.003) as independent POPF risk factors. A combination of CRP and drain amylase was effective in the early detection of CR-POPF after PD.

Ansorge [43] hypothesized that ischemia and local proteolytic enzyme activation in the proximity of pancreatic anastomosis (changes also described in acute pancreatitis) form a potential mechanism of POPF. The authors applied intraperitoneal microdialysis to monitor the metabolic changes and protease activation intra-abdominally after PD. The following parameters were considered: lactate-to-pirogroniane ratio (L/P), which is a marker of tissue hypoxia, trypsinogen-activating peptide (TAP), which is detached from trypsinogen when it activates, and glucose levels. In 7 of 48 patients after PD, POPF was found. This group had a significant elevation of L/P on POD1–5 and elevated serum amylase in comparison with other patients. Intraperitoneal elevated TAP (>0.1 μg/L) on POD1 was found in 6 of the 7 POPF patients, compared to 2 of 33 patients without complications. The authors concluded that these changes resulted from trypsin and lipase activation, as the first step in POPF formation caused by ischemia and decreased tissue perfusion, leading to pancreatic necrosis. Therefore, a concept of local cell ischemia accelerating POAP development was formed. The intraperitoneal lactate concentration was higher than the systemic concentrations in all patients on POD 1–5 (*p* < 0.001). In POPF patients, elevated intraperitoneal lactate was not accompanied by systemic hyperlactate. The intra-abdominal samples were more sensitive to pancreatic inflammation and tissue ischemia than the serum samples.

In conclusion, monitoring of the drain fluid seems to be a useful tool in early POPF diagnosis [44] and possibly also for POAP detection. However, there is no consensus on the upper threshold of drain amylase activity for the diagnosis of POAP and/or POPF. The 2022 ISGPS definition for postpancreatectomy acute pancreatitis does not include drain amylase, using it only for POPF diagnosis. It is worth noting that many authors recommend a fast postoperative track with early drain removal if the pancreatic enzymes are low in the fluid [29,37,45].

### 4.5. Abdominal CT

Rudis [6] performed abdominal and pelvic CTs in 14 patients with POPF grade C. In 4 of these patients, POAP was found during autopsy. CT performed in these patients before surgical revision showed postoperative changes, fluid collections, and contrast leakage from the anastomosis, but in none of the cases was pancreatitis diagnosed. Therefore, the authors concluded that CT does not necessarily distinguish features characteristic of POAP. Fatty tissue enhancement and inflammatory changes in the postoperative site and around the pancreatic remnant are difficult to differentiate from postoperative changes, especially in mild pancreatitis [26]. Imaging results must always be confronted with the clinical pancreatitis symptoms. More evident inflammation and fluid collections around the pancreatic stump than in the site of the removed specimen, without a tendency to spread in the retroperitoneal space, suggest POAP rather than postoperative changes [46,47], as does abnormal thickening of the prerenal fascia [26,47].

In the study by Räty [48], POAP was diagnosed based on CT, which was routinely performed on POD 2 and 5, and also later if there were any clinical indications. Focal or general enlargement, irregular outline, irregular enhancement of the pancreatic stump, and inflammatory changes in the upper left suprarenal region were assessed. Out of ten patients with POAP, in six it was detected on POD2, but in the remaining four patients it was diagnosed by CT on POD5. As CT detects only severe pancreatitis cases, the incidence of POAP was probably higher than the 27% (10/37) reported by Räty [47].

In the previously mentioned work by Partelli [31], apart from the serum amylase assessment, radiological features of CR-POAP and CR-POPF were also studied. The patients had a postoperative contrast-enhanced CT (CECT). In the group with CR-POAP, only 25% of the patients who had CECT showed characteristic radiological signs of pancreatitis. It is important to note that this number would probably be higher, as not all patients with elevated serum amylase and CRP had postoperative CECT, and abdominal fluid collections were not considered a radiological POAP sign because of the difficulty in differentiating from POPF. Additionally, patients with benign acute pancreatitis do not present any radiological changes. In Partelli’s study, the radiological signs of acute pancreatitis were more pronounced in the group of patients with amylase activity > 300 U/L and CRP 180 mg/L, but in 9 of these 22 patients, CR-POPF did not occur.

Loos [32] differentiated hyperamylasemia from POAP based on radiological criteria. CECT was performed postoperatively in patients with a worsening clinical condition. POAP was classified according to the CT severity as (0) lack of pancreatitis, (1) benign, (2) moderate, and (3) severe pancreatitis. CT showed typical POAP features in 103 (28%) of patients. Of these, radiologically benign POAP was found in *n* = 55; 53.4% of patients, moderate in *n* = 34; 33%, and severe in *n* = 14; 13.6%. Two hundred sixty-one patients (72%) did not have radiological POAP features. In 364 patients who had postoperative CT performed, in 242, hyperamylasemia was found on POD1: in 102 (42%), radiological POAP was also found, and in 140 (58%), it was not. The author concluded that although amylase and CRP in serum are useful in the early postoperative course to identify or exclude the risk of POAP, the biochemical analysis alone is insufficient. CT (or other imaging tests) are required to confirm the presence of POAP.

Another interesting imaging method was examined by Sugimoto [49], who claimed that CT perfusion (CTP) of the pancreas facilitates precise preoperative POPF risk assessment. It may also prove useful for POAP because both complications are closely related. The method enables the examination of organ hemodynamics. In 20 patients, CTP was performed before PD, and a correlation between CTP parameters and POPF was found with high specificity and predictive value.

It is worth noting that Bannone et al. recently performed a study on 65 patients after PD to evaluate the usefulness of diffusion-weighted magnetic resonance imaging in POAP diagnosis; however, they do not recommend this method for standard use [50].

Table 3 presents CT features in POAP according to the available literature. The features listed below, visible in contrast-enhanced CT, offer useful diagnostic insights, have high sensitivity but low specificity, and should be considered in conjunction with laboratory test results and the patient’s general condition. It was recently shown that Diffusion-Weighted Imaging (DWI), which provides functional information on tissue properties, considerably improves the diagnostic accuracy in POAP. Increased cellularity and interstitial edema are indicative of pancreatic inflammation, as is a hyperintense signal on DWI with decreased apparent diffusion coefficient values [51].

Also in our previous study [52], diffuse or localized inflammatory enlargement of the pancreatic stump, inflammatory changes in the peripancreatic fat tissue, peripancreatic fluid collections, necrosis of the pancreatic parenchyma, peripancreatic necrosis, leakage of pancreatic anastomosis, peritonitis, and bleeding were significantly related to the development of POAP (*p* < 0.001).

**Table 3:** POAP features in CT [26,31,48,51].

CT Features in POAP	
pancreatic stump and peripancreatic fat inflammation	→ edema [32] → enlargement → irregular contour → irregular enhancement → fat stranding [51] → necrosis
thickening of the anterior perirenal fascia and fluid collections in this localization [26,47]	
anastomotic leakage [46,47]	→ contrast leakage from the anastomosis—certain anastomotic dehiscence → large amount of fluid around the anastomosis, fluid collections, abscesses in the proximity of the anastomoses—indirect proof of anastomotic dehiscence
ileus/subileus—intestinal distension	
signs of former or active bleeding [53]	→ hematomas → clots

### 4.6. Clinical Features

POAP assessment in the early stage is very challenging—the symptoms may be subdued by analgesics and mechanical ventilation. Rudis [6] points out that an irregular postoperative course, with abdominal pain, bloating, subileus, and cloudy abdominal drainage, may suggest developing POAP.

The first alarming sign may be progressing circulatory failure, especially when blood supplementation is required (which, however, can also be caused by other postoperative complications).

Patients with POANP distinguished by Globke [28] most often do not show postoperative progress but remain suffering. POANP is connected to generalized inflammatory response, multiorgan failure, immunosuppression, and organ damage. POANP is also connected to a higher prevalence of other severe postoperative complications and prolonged hospital stay. CRP, APACHE II and SOFA correlate with the severity of the postoperative condition and may predict the POANP: patients after total pancreatectomy because of POANP had significantly higher APACHE II and SOFA median score on POD 4 after PD (9 (4–15) vs. 5 (5–12) *p* < 0.001) and (4 (2.5–8) vs. 2 (2–4) *p* < 0.001), on the day before total pancreatectomy (10 (7–14) vs. 4 (3–8), *p* < 0.001) and (4 (1–7) vs. 2 (1–3), *p* < 0.001), on the reoperation day (11 (5–19) vs. 6 (3–10), *p* < 0.001) and (4 (12–6) vs. 2 (1–4), *p* < 0.001), and on the day after pancreatectomy (12 (7–14) vs. 7 (4–9), *p* < 0.001) and (5 (4–6) vs. 2 (1–3), *p* < 0.001).

Räty [48] showed that POAP is one of the main causes of delayed gastric emptying (DGE) after PD. DGE is found in 20–40% of the patients after pancreatic surgery, especially after PD. It is a complication caused by multiple factors, including lack of hormonal stimulation (motilin) after duodenal resection, ischemia of the pylorus after right gastric artery, gastroduodenal artery ligation, gastric innervation after radical removal of the surrounding tissues, and it can also be secondary to intra-abdominal complications [54]. Räty examined 39 patients after PD, and 12 developed DGE. Surgical complications (9/12 vs. 5/27, *p* = 0.001) were more prevalent in the DGE group. POAP (6/12 vs. 4/27, *p* = 0.03) and POPF (5/12 vs. 1/27, *p* = 0.0007) diagnosed in CT performed on POD 2 and 5, had a significant correlation with DGE. In the group of patients with DGE, the blood parameters showing significant changes were serum amylase (average +/− SEM 715 +/− 205 vs. 152 +/− 70 IU/50; *p* = 0.02), blood leukocytes (9 vs. 16 +/− 2 +/− 0.6 × 10^9^/50; *p* = 0.007), and CRP (144 +/− 28 vs. 51 +/− 14 mg/50; *p* = 0.01). POAP may be an important DGE-inducing factor. Therefore, all patients with DGE should be carefully observed for POAP development. The clinical symptoms of POAP are summarized in Table 4.

### 4.7. Diagnostic Algorithm for POAP

**Figure 2:**
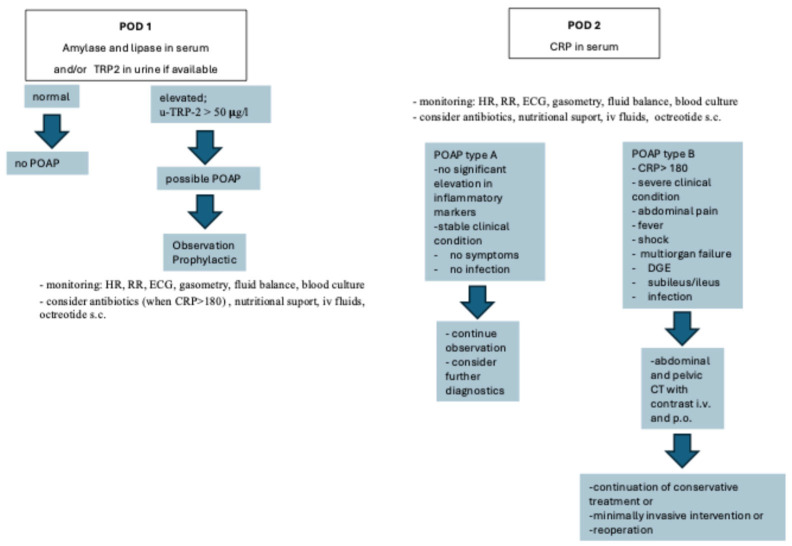
Suggested diagnostic algorithm for POAP. Abbreviations: POAP—postoperative acute pancreatitis, POD—postoperative day, u-TRP—urine trypsinogen-2, HR—heart rate, RR—arterial blood pressure, ECG—electrocardiograph, CRP—C-reactive protein, DGE—delayed gastric emptying, CT—computed tomography.

Based on the available data as presented above, in Figure 2, we propose a diagnostic algorithm for POAP that might enable its early diagnosis. It is worth noting that although there are u-TRP fast strip tests, to our knowledge, they are not widely available. However, we included u-TRP as a possibility in the proposed algorithm as it has high specificity and sensitivity in acute pancreatitis diagnosis.

## 5. POAP Consequences

The study by Ikenaga [21] analyzed 153 patients after PD and 166 patients after DP. Both POAP and CR-POAP increased the risk of severe PD complications, but after DP, only CR-POAP, and not POAP itself, was associated with severe complications; therefore, the clinical significance of POAP after DP may be low. After DP, CR-POAP caused a higher incidence of severe complications (Clavien-Dindo ≥ IIIA; *p* = 0.0067), POPF (*p* = 0.0307), and abdominal abscesses, however, with no impact on hospital stay.

In patients who developed POAP after PD, increased severe complication rates (Clavien-Dindo ≥ IIIA) were observed, including higher rates of POPF and abdominal abscesses. The hospital stay of these patients was longer (median 21 vs. 17 days). 37 of 97 patients (38.1%) with POAP had CRP above 180 mg/L on POD3 (CR-POAP). Also, CR-POAP after PD had a significantly higher incidence of severe complications (Clavien-Dindo ≥ IIIA, 34.4% vs. 12.4%; *p* = 0.0032) and longer hospital stay (median: 36 vs. 18 days; *p* < 0.0001). CR-POAP was an independent risk factor for POPF after PD.

Andrianello [25] showed that patients with POAP after DP had significantly more severe complications (Clavien-Dindo ≥ III), POPF, hemorrhage, abdominal abscesses, sepsis, and relaparotomies. They were significantly more often discharged with abdominal drainage sustained and had more bacterial infections, including multidrug-resistant bacteria in the drained fluid. The occurrence of POAP predicted severe complications (Clavien-Dindo ≥ III) with 81% sensitivity, 34% specificity, 18% PPV, and 91% NPV. CR-POAP had 66% sensitivity, 59% specificity, 90% NPV, and 23% PPV. When amylase levels higher than three times normal were used as a criterion for POAP, 28% sensitivity, 90% specificity, 87% NPV, and 35% PPV were found. Of patients with POAP (*n* = 250), 93 (37.2%) developed POPF. When only patients with CR-POAP (*n* = 166) were considered, POPF was found in 72 (43.3%).

Comparing the patients who did not have POAP or POPF with those who only had POAP (*n* = 157), the latter group had significantly more biochemical leaks (former POPF A) and bacterial infection in the drain fluid. Patients who had POPF with or without POAP had more severe complications (Clavien-Dindo ≥ III), hemorrhage, DGE, abscesses, sepsis, cardiac morbidity, pneumonia, bacterial infections in the drain fluid, and relaparotomies. They also needed intensive care more often and had a higher readmission rate.

In Chen’s analysis of PD patients [24], CR-POPF was more prevalent in patients with POAP (28.70% vs. 8.06%, *p* < 0.001). Patients with POAP also had other complications: bile leakage, abdominal infections, abscesses, and reoperations. POAP required longer abdominal drainage and a longer hospital stay.

Winter [55] performed a retrospective analysis of 2323 patients after PD. Postoperative amylase ≥ 292 U/L was an important predictor of postoperative complications, most prominently POPF. POPF rates in groups with postoperative amylase activity measured as low (0–99 U/L), medium (100–399 U/L), and high (≥400 U/L) were 4%, 14%, and 20%, respectively.

Apart from POPF, elevated amylase is also connected to an increased risk of DGE [36,45,48,55], intra-abdominal abscesses [45,55], and postoperative mortality [18,55].

In the large study by Loos [32], patients with POAP had higher morbidity and mortality. POPF was diagnosed in 32.4% of this group; hemorrhage in 14.7%; reoperation in 29.4%; total pancreatectomy had to be performed in 9.8%, and 90-day mortality was 11.8%.

Yoo’s work stands out due to contradictory results [56]. A total of 246 patients were analyzed, and in 191 (77.6%), POAP after PD was found according to Connor’s criteria. The study did not find significant differences in postoperative complications in general (*p* = 0.257), in severe complications (Clavien-Dindo ≥ IIIA) (*p* = 0.333), CR-POPF (*p* = 1.000), DGE (*p* = 0.685), and hemorrhage (*p* = 1.000) between patients with and without POAP. Neither POAP nor CR-POAP was a significant CR-POPF predictor in one-factorial or multifactorial analysis (OR 0.998, 95% 101, 0.310–3.886; *p* = 0.998).

Based on the available literature, it seems clear that POAP is an important clinical problem, directly associated with postoperative complications in pancreatic surgery.

## 6. POAP Prevention

There are no separate guidelines on POAP prevention. As POAP seems to be an important cause of POPF, POPF prevention may also help prevent POAP. Based on POAP pathophysiology, the assessment of pancreatic stump perfusion appears to be of greatest importance.

The blood supply of the pancreatic stump can be assessed intraoperatively using indocyanine green (ICG), similar to the method used for laparoscopic intestinal resections [57,58]. Prior to pancreaticojejunal anastomosis, the pancreatic stump’s viability is assessed with indocyanine green administered intravenously and with infrared light. Right before the surgeon proceeds with pancreatico-jejunal anastomosis, the anesthesiologist administers 2 mL (0.5 mg) of ICG (concentration 0.25 mg/mL) intravenously. The pancreatic margin is observed in an infrared camera. The ICG enhances the well-perfused tissue. Identifying the ischemic pancreatic margin allows for adjusting the margin and achieving a well-perfused stump. This may lower the risk of POPF. Cutting of the isthmus of the pancreas may cause pancreatic ischemia because it is where the blood supply from the pancreatic head and the trunk connects [7,57]. A more distal cutting line may be beneficial for the perfusion of the anastomosis, as shown by Strasberg [59]. In his study, an ultrasound Doppler examination of the pancreatic stump proved useful for blood supply assessment, and a more distal cutting line chosen for stumps with insufficient vascularization led to a lower incidence of POPF. The author insists that the optimization of the anastomotic perfusion and meticulous surgical technique allows for a significant reduction in POPFs after PD.

Considering the anastomotic techniques, we did not find studies focusing on POAP and anastomotic techniques per se; however, multiple studies analyze the anastomoses regarding POPF occurrence. Some meta-analyses showed that pancreaticogastric anastomosis seems to be associated with a lower incidence of CR- POPF [60,61]. Other authors, however, in their meta-analyses did not find significant differences in POPF occurrence depending on the anastomotic technique, although it is worth mentioning that the available studies vary regarding surgical techniques and POPF definition, which limits the conclusiveness of the analyses [62,63]. Among the pancreaticogastric anastomosis advantages are inactivation of pancreatic enzymes by gastric acid, the relatively easy surgical technique, and low risk of ischemia and tension [61].

In Table 5, we summarize the potential possibilities for POAP prevention.

## 7. POAP Treatment

No study has yet defined any specific treatment strategy for POAP. The only treatment available at present is the same as acute pancreatitis treatment, administered early and with proper monitoring of the clinical condition. Fluid therapy is crucial, with lactated Ringer’s solution being the initial choice. Nowadays, moderate fluid resuscitation is recommended, with an infusion rate of 1.5 mL/kg/h [67].

In POAP, infection control is extremely important. Antibiotics are administered to manage any proven infection. The most commonly used are broad-spectrum antibiotics—carbapenemes or fluoroquinolones are often considered first-line treatment due to their ability to penetrate pancreatic tissue. Alternatively, third-generation cephalosporine with metronidazole is an option [68,69].

Nutrition in patients with POAP can involve oral, enteral, or parenteral nutrition. The choice of feeding method depends on the individual’s condition, the severity of pancreatitis, and the patient’s condition. Oral nutrition is reserved only for asymptomatic patients— it can start with clear liquids and gradually progress to solid food. Enteral nutrition through a nasojejunal tube is often preferred due to its potential to improve immune function and reduce sepsis risk. Parenteral nutrition is sometimes required, but it carries a higher risk of complications and is generally reserved for cases where enteral feeding is not feasible or tolerated [67,70]. Analgesia is achieved with non-steroidal anti-inflammatory drugs and/or opioids.

Sandostatin (octreotide), a somatostatin analog, is sometimes used in the management of postoperative pancreatitis, particularly in the context of pancreatic fistulas. It works by reducing pancreatic exocrine secretions, which can help to reduce the output from fistulas and potentially promote their closure. In a meta-analysis by Alghamdi et al., perioperative octreotide significantly reduced POPF incidence, but without a significant difference in postoperative mortality [71]. Prophylactic administration of ulinastatin was also shown to reduce the amylase concentration in serum and in drains as well as POAP occurrence [41]; however, none of these drugs are universally recommended for POAP or POPF treatment and prophylaxis. The data on octreotide is not consistent, and some studies do not confirm its beneficial effects [72,73,74].

For extensive necrosis and/or fluid collections, an endoscopic drainage is now most often recommended, with a step-up approach to more invasive intervention, which is surgery [67]. Surgical management can involve the following:

- Debridement of the necrotic tissue and drainage;
- Revision of the pancreatic anastomosis—in cases of anastomotic dehiscence, redoing the pancreaticojejunostomy (PJ) anastomosis may be necessary [67,69,75,76,77].

In the study by Rudis [6], all four patients with POAP died. The author concluded that POAP with POPF grade C coexistence almost always leads to death. According to the study, drainage and removal of pancreatic anastomosis is not sufficient, and the optimal though risky approach is early revision with complete pancreatic resection and splenectomy. In late revisions, the inflammation much changes the operative field, and these reoperations have high mortality. Total pancreatectomy can be beneficial immediately after diagnosing the potentially mortal POAP [75]. In some cases, upfront total pancreatectomy is a method to avoid potentially fatal PPAP. It is accepted in selected cases with a higher risk of pancreatic anastomosis leakage due to soft pancreatic tissue and a narrow MPD. However, in the absence of other indications (e.g., disease affecting the entire gland), there are no uniform recommendations, and the decision is made by the surgeon [78].

Total pancreatectomy has high mortality and morbidity—in a recently conducted meta-analysis [79], the mortality rate was 44.5%, and the morbidity rate was 72.6%, but despite these poor results, total pancreatectomy is performed as a salvage procedure [76].

In our previous study involving 428 patients after PD surgery, a total pancreatectomy was performed in 22 patients, with 20 of them due to PPAP (*p* < 0.0001)—this type of reoperation is therefore specific to this complication [52]. Additionally, there are reports [80] that postoperative hyperamylasemia on POD 1 was identified as an independent risk factor of complete pancreatectomy.

Recent research emphasizes that only very few patients (3%) need completion pancreatectomy [81]. Authors suggest that conservative, interventional, and organ-preserving surgical measures are the mainstay of complication management after pancreatic operations. Postpancreatectomy acute necrotizing pancreatitis is one of the situations (next to uncontrollable postoperative pancreatic fistula and fistula-associated hemorrhage) that is highly dangerous and represents the main indications for completion pancreatectomy, but eventually, the choice of methods of the surgical treatment depends on the patient’s individual condition and the surgeon’s discretion.

## 8. Conclusions

Despite decades of intensive studies focused on pancreatic surgery techniques, POPF incidence after pancreatic resections remains relatively unchanged. POAP development in the early postoperative course seems to be a trigger for POPF. Despite expanding knowledge on POAP pathophysiology, we still lack universal guidelines on its diagnosis, prevention, and treatment. According to available studies, it seems justified to routinely monitor serum amylase and lipase on POD1, and CRP in serum and amylase in drain fluid on POD2 to detect POAP early. Multidisciplinary studies and coordination of the available diagnostic and therapeutic methods are needed to provide progress and reduce complication rates in pancreatic surgery.

## Figures and Tables

**Table 1:** POAP risk factors depending on the operation type. PD—pancreatoduodenectomy, DP—distal pancreatectomy [21,22,23,24,25].

Type of Operation	POAP Risk Factors
PD	Narrow main pancreatic duct—diameter < 3 mm
	Diagnosis other than adenocarcinoma
	Normal preoperative bilirubin level
	Female sex
	Serum amylase activity
	Soft pancreatic tissue
	Longer operative time > 295 min
	Preoperative interleukin-6 levels
	Soft pancreatic tissue
	Robotic surgery
DP	Pancreatic thickness > 9.5 mm
	ASA ≥ 2
	Pancreatic tissue sparing
	Neuroendocrine tumors
	Age < 65
	Neoadjuvant therapy (lower risk)
	Dilated MPD (lower risk)

**Table 2:** Connor’s POAP classification [7]. Abbreviations: POAP—postoperative acute pancreatitis, u-TRP—urine trypsinogen-2, POD—postoperative day, CRP—C-reactive protein [27].

Connor’s POAP Classification	
u-TRP > 50 ug/L (POD 1–2)	Yes
Serum amylase/lipase activity > normal (POD 0–1)	Yes (if u-TRP unknown)
Elevated amylase activity in drain >3 xnormal (POD 3)	Yes/No
CRP < 180 mg/L (POD 2)	POAP without clinical significance
CRP > 180 mg/L (POD 2)	Clinically relevant POAP
No symptoms, no infection, no specific therapy needed	Grade A
POAP with symptoms or specific therapy needed, including interventional therapy	Grade B
POAP with reoperation or mortal without reoperation	Grade C

**Table 4:** Clinical symptoms of POAP. Abbreviations: POAP—postoperative acute pancreatitis, DGE—delayed gastric emptying, POPF—postoperative pancreatic fistula.

Clinical Symptoms Suggesting POAP	
abdominal pain	
DGE (nausea, vomiting)	
ileus/subileus symptoms	→ cloudy fluid—suggests POPF → bleeding—sentinel bleed—may precede hemorrhage due to POPF
abnormal abdominal drainage	
fever	
septic shock	

**Table 5:** POAP potential mechanisms and possibilities of their prevention. Abbreviations: PD—pancreatoduodenectomy, DP—distal pancreatectomy, MPD—main pancreatic duct, NET—neuroendocrine tumor, PDAC—pancreatic ductal adenocarcinoma, POAP—postoperative acute pancreatitis, BMI—body mass index, ASA—American Society of Anesthesiologists.

ETIOLOGY	MECHANISM		PREVENTION
ISCHEMIC	SURGICAL		
	Higher risk	Lower risk	→ extremely careful pancreatic tissue handling, avoiding of pulling, pressing and crushing → meticulous anastomotic technique, no ischemia or tension in the anastomosis → safe and effective hemostasis → using modern and safe surgical tools
	Non-modifiable risk factors: → PD [23]	Non-modifiable risk factors: → DP [23]	
	Modifiable risk factors: → pancreaticojejunal anastomosis? [60,61,62,63] → parenchyma—saving DP [25] → robotic PD [24] → vascular resection [23] → higher blood loss [21,23,24] → MPD obstruction [37,43] → line of cutting in the pancreatic isthmus [57] → mechanical damage to pancreatic tissue through extensive manipulations [37]	Modifiable risk factors: → pancreaticogastric anastomosis? [60,61,62,63] → extended DP [25] → pancreatic stump reduction [18,59]	
	ANESTHESIOLOGICAL		→ appropriate fluid administration → extrameningeal anesthesia
	Modifiable risk factors: → too few intraoperative fluids [18] → no extrameningeal anesthesia [64]		
	ANATOMICAL		→ thorough preoperative assessment of pancreatic vasculature in CT or angiography
	Non-modifiable risk factors: → vascular anomalies [7] → pancreatic duct anomalies [23]		
HISTOLOGICAL	Higher risk	Lower risk	No prevention
	Non-modifiable risk factors: → ampullary cancer → duodenal cancer → cystic tumor → lobular carcinoma → NET	Non-modifiable risk factors: → PDAC → chronic pancreatitis [23,24,25,29]	
MORPHOLOGICAL	Pancreatic features with higher risk of POAP		Precise preoperative determination of the POAP risk factors, MPD diameter and pancreatic tissue consistency
	Non-modifiable risk factors: → soft tissue [18,21,29,48,55,65] → high concentration of lobular cells in the pancreatic tissue [37] → higher thickness of the pancreas [25] → small (<3 mm) MPD diameter [18,21,24,29,65] → pancreas divisum [66]		
CLINICAL	Higher risk	Lower risk	Accurate history and physical examination leading to an individual therapeutic plan
	Modifiable risk factors: → high BMI [24,65] → long operative time (>8 h) [6,21,24]	Modifiable risk factors: → neoadjuvant treatment [18,25] → presence of biliary stent [23]	
	Non-modifiable risk factors: → ASA > 2 [21] → coronary disease? [48] → preoperative jaundice [24] → female sex? [24] → male sex? [56]	Non-modifiable risk factors: → coronary disease? [23] → exocrine insufficiency → age over 65 years old [25]	

## Data Availability

The original contributions presented in this study are included in the article. Further inquiries can be directed to the corresponding author(s).

## Abbreviations

The following abbreviations are used in this manuscript:

APACHE II	Acute Physiology and Chronic Health Evaluation II
ASA	American Society of Anesthesiologists
AUROC	Area under the receiver operating characteristic
BMI	Body mass index
CECT	Contrast-enhanced CT
CRP	C-reactive protein
CR-POAP	Clinically relevant POAP
CR-POPF	Clinically relevant POPF
CT	Computed tomography
CTP	CT perfusion
DGE	Delayed gastric emptying
DP	Distal pancreatectomy
DWI	Diffusion-Weighted Imaging
ECG	Electrocardiograph
GPAR	Gastric/pancreatic amylase ratio
HR	Heart rate
ISGPS	International Study Group for Pancreatic Surgery
MRI	Magnetic resonance imaging
NET	Neuroendocrine tumor
NPV	Negative predictive ratio
PD	Pancreatoduodenectomy
POANP	Postoperative necrotic pancreatitis
POAP	Postoperative acute pancreatitis
POD	Postoperative day
POH	Postoperative hyperamylasemia
POPF	Postoperative pancreatic fistula
PPAP	Postpancreatectomy Acute Pancreatitis
PPV	Positive predictive ratio
RR	Arterial blood pressure
SOFA	The Sequential Organ Failure Assessment
TAP	Trypsinogen-activating peptide
u-TRP	Urine trypsinogen-2
